# An Explicit Test of Kill the Winner: Protistan Grazing and Phage Lysis Differentially Impact Fast‐Growing Bacterial Taxa in the Coastal Antarctic

**DOI:** 10.1111/1462-2920.70254

**Published:** 2026-02-09

**Authors:** Elizabeth Connors, Abigail Coker, Grace S. Wang, Lisa Zeigler, Jeff S. Bowman

**Affiliations:** ^1^ Scripps Institution of Oceanography, UC San Diego La Jolla California USA; ^2^ Scripps Polar Center, UC San Diego La Jolla California USA

## Abstract

Protists and bacteriophages exert top‐down control on bacterial populations. Previous work in the coastal Antarctic demonstrates the potential for intra‐seasonal variability of this top‐down control driven by the extreme seasonal contrast in bacterial growth rates. We evaluated whether predators ‘kill the winner’ wherein protists and phages preferentially impact the most abundant members of bacterial assemblages over an austral summer with weekly dilution experiments. Seawater from 10 m was divided into two serial dilutions with either 0.2 μm (to evaluate protist grazing) or 30 kDa (to evaluate protist grazing and lysis from bacteriophage) filtered water. We observed strong intra‐seasonal change of bacteriophage and protistan contributions to mortality. A comparison of activity per ASV from amplicon sequencing over our dilution experiments to a predicted minimal doubling time indicates that ‘kill the winner’ is occurring during the top‐down control of only a few bacteria. As not all bacterial taxa with a predicted low mean doubling time demonstrated high activity in our dilution experiments, our results indicate protists and phage selectively target some fast‐growing or abundant bacteria which we term ‘kill select winners’ (KsW). Overall, our evaluation of bacterial abundance and community structure provides unprecedented knowledge of top‐down control of marine bacteria.

## Graphical abstract

We evaluated whether predators ‘kill the winner’ wherein protists and phages preferentially impact the most abundant members of bacterial assemblages over an austral summer with weekly dilution experiments. Seawater from 10 m was divided into two serial dilutions with either 0.2 μm (to evaluate protist grazing) or 30 kDa (to evaluate protist grazing and lysis from bacteriophage) filtered water. The difference between these two treatments, calculated by fitting a linear model to bacterial abundance data, indicates strong intra‐seasonal change of bacteriophage and protistan contributions to mortality.

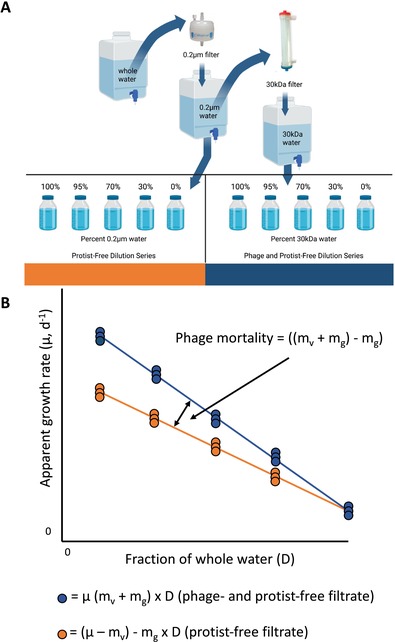

## 1 Introduction

Heterotrophic marine bacterioplankton (bacteria and archaea) are both a key component of the marine food web and important regulators of the global carbon cycle (Worden et al. 2015). Marine bacteria repackage dissolved organic carbon derived from primary production into biomass, making this carbon available for bacterivorous protists and ultimately higher trophic levels in a process known as the microbial loop (Azam et al. 1983). The strength of this microbial loop in the surface ocean in part regulates the efficiency of carbon export; a strong microbial loop retains carbon in the surface ocean and a weak microbial loop means more carbon can be exported out of the surface ocean (Legendre and Rassoulzadegan 2012). Complicating the fate of bacterial carbon in the microbial loop are bacteriophage, viruses that can in certain cases lyse bacterial biomass, returning it to dissolved organic carbon in a process known as the viral shunt (Wilhem and Suttle 1999). Taken together, bacterivorous protists and bacteriophage exert strong top‐down control on bacterial biomass and can modulate the efficiency of carbon export from the surface ocean.

Both bacteriophage and bacterivorous protists are thought to preferentially impact the most active members of the bacterial assemblage (Thingstad and Lingnell 1997), a concept termed ‘kill the winner’ (KtW). Critically, as bacterioplankton compete for limited resources in marine environments, this selective loss of competitive populations prevents ‘winning’ populations from sequestering all of the resources and allows more defensive populations to coexist alongside them, maintaining microbial diversity (Winter et al. 2010). The magnitude of KtW predation is therefore intrinsically linked to resource availability in each environment, which determines bacterial growth rates, biomass accumulation and ultimately predation rates of competitive populations. While our theoretical understanding of KtW has expanded in recent years (Maslov and Sneppen 2017; Xue and Goldenfeld 2017; Zhong et al. 2023), environmental observations of this important ecological phenomenon in dynamic systems remain very sporadic (Winter et al. 2010; Yang et al. 2023).

The waters off the western Antarctic Peninsula (WAP) are an ideal model system for testing KtW and exploring bacterial mortality dynamics. The WAP marine microbial community is highly responsive to dramatic changes in environmental conditions, shifting from a largely chemolithoautotrophic/oligotrophic community during winter darkness (Bowman et al. 2017; Dutta et al. 2023) to a heterotrophic/copiotrophic community in response to high biomass blooms of phytoplankton during the sunlight summer months (Luria et al. 2016; Bunse and Pinhassi 2017). The subsequent grazing of this rapid microbial growth by bacterivorous protists has both been modelled and observed in the western Antarctic Peninsula region, where grazing rates can exceed bacterial uptake of carbon on occasion (Bird and Karl 1999; Garzio and Steinberg 2013; Ducklow et al. 2015). In separate studies, bacteriophage have been shown to be tightly coupled to bacterial populations, with increased lytic phage activity during the early summer (Guixa‐Boixereu et al. 2002; Brum et al. 2015; Evans et al. 2017).

In this study we first quantify the changes in microbial cell abundances and community composition during the austral summer season (December to March), with twice weekly measurements for flow cytometry and for amplicon sequencing (16S and 18S rRNA gene sequencing) over two field seasons. We then performed a weekly dilution experiment of two dilution series to measure and predict predation pressure from bacterivorous protists (henceforth protists) and bacteriophage (henceforth phages). Critically, we included measurements for community composition (16S rRNA gene sequencing) in every dilution experiment to measure KtW directly for each bacterial amplicon sequence variant (ASV) by comparing ASV mortality rate to a predicted bacterial doubling time. We designed our study to improve our understanding of top‐down control of heterotrophic bacteria in polar seas, and to examine how KtW manifests in a natural microbial community over time.

## 2 Materials and Methods

A CTD (Seabird SBE19v2) and rosette were deployed from the small research vessel *Hadar* at Station E (64.815° S, 64.0405° W) twice weekly during the austral summers, respectively from December 2022 to March 2023 (PAL2223) and from November 2023 to March 2024 (PAL2324) to measure changes in temperature and to collect seawater for analysis. Seawater was collected with Niskin bottles on the rosette from three depths (10, 30 and 50 m). While water from these depths was used for flow cytometry and amplicon sequencing, only 10 m water was used in the induction and dilution experiments and in our analyses below. Minimum air temperature, wind speed and direction from nearby Palmer Station (64.7743° S, 64.0538° W), Antarctica were downloaded with the weather and climate access tool Meteostat (Lamprecht 2024) for both summer seasons.

### 2.1 Flow Cytometry

Flow cytometry was used to count abundances of phytoplankton, bacteria, viral like particles (VLP), mixotrophic and heterotrophic eukaryotes and bacteria with high rates of respiration. A subset of the flow cytometry samples was run immediately at Palmer Station on an AccuriC6 flow cytometer (BD Biosciences, USA) which interrogates cells with a blue laser (488 nm). These live samples included unstained (for autofluorescence, AF), Lysotracker Green (Thermofisher, USA) stained (for mixotrophic phytoplankton, LSG) and BacLight Redox Sensor Green (Thermofisher, USA) stained (for bacteria with high cellular activity, RSG). Quality control for absolute cell counts was confirmed by adding 10 μL of 1:2500 diluted 1 μm Fluoresbrite Yellow Microspheres (Polyscience Inc.) to a 1 mL aliquot of each sample. AF samples were run on ‘fast’ with a flow rate of 66 μL min^−1^ for 3 min, and measured for forward scatter, side scatter and both red (488/675 nm excitation/emission) and yellow emission (488/585 nm excitation/emission).

For the live stained samples, 1 μL of 50 nM Lysotracker stain was added and for the RSG stained samples, 1 μL undiluted RSG stain was added to 1 mL samples, respectively. Both types of live stained samples were then incubated in the dark for 15 min at 4°C. LSG‐stained samples were run on ‘fast’ with a flow rate of 66 μL min^−1^ for 3 min and measured for forward scatter, side scatter and both red (488/675 nm excitation/emission) and green fluorescence (488/533 nm excitation/emission) and were taken only for the PAL2223 season (December 2022 to March 2023). RSG samples were run on ‘slow’ with a flow rate of 13 μL min^−1^ for 1 min and measured for forward scatter, side scatter and green fluorescence (488/533 nm excitation/emission), and were taken for the full duration of the two summer seasons.

The remaining flow cytometry samples, including those collected during the dilution experiments, were fixed with 10 μL of 25% glutaraldehyde per mL to a final concentration of 0.25% for 15 min at 4°C and frozen at −80°C until they could be run on a Guava flow cytometer (for SG stained samples) or a FACS Aria flow cytometer (for VLP samples). SG samples were stained and incubated in the dark for 15 min with the nucleotide stain SYBR Green 1 (Molecular Probes, USA) diluted at the manufacturer's recommended concentration. These samples were run on the Guava with a flow rate of 14 μL min^−1^ for 1 min and measured for forward scatter, side scatter and green emission (488/525 nm excitation/emission) spiked with 10 μL 1:10 diluted 123easycheck beads (Thermofisher, USA). For the VLP samples, 10 μL sample was diluted in sterile 130 μL 1× PBS and then were stained and incubated in the dark for 15 min with the nucleotide stain SYBR Green 1 (Molecular Probes Inc.) diluted 2000×. Diluted and stained VLP samples were run on the FACS Aria with a flow rate of 35 μL min^−1^ for 1 min. Events were measured for forward scatter with a photomultiplier (FSC‐PMT), enabling discrimination of a range of 0.05–0.5 μm beads (Flow Cytometry Sub‐Micron Particle Size Reference Kit, Thermofisher, USA), and green emission (488/533 nm excitation/emission).

For all flow cytometry samples, populations were identified using a self‐organising map (SOM) from excitation and emission following previous methods (Bowman et al. 2017; Wilson et al. 2021). In brief, five randomised samples were selected to construct a training set for each type of sample. These data were then trained using a toroidal map with a grid size of 41 × 41 with the ‘kohonen’ package in R (Wehrens and Kruisselbrink 2018). Populations were identified using *k*‐means clustering with *k* = 4 for AF samples, *k* = 5 for LSG samples, *k* = 4 for RSG samples, *k* = 4 for SG samples and *k* = 4 for VLP, which was then used to classify events in all flow cytometry samples.

Three clusters were identified as high chlorophyll (high Chl), low chlorophyll (low Chl) and high phycoerythrin (high PE) in the AF model; two clusters were identified as high LSG/high Chl (mixotrophs) and high LSG/low Chl (heterotrophs) in the LSG model; two clusters were identified as high RedoxSensor Green (high RSG) and low RedoxSensor green (low RSG) in the RedoxSensor Green model; two clusters were identified as high nucleic acid (HNA) and low nucleic acid (LNA) clusters in the SG model and one cluster was identified as viral like particles (VLP) in the VLP model. These clusters were converted into cells mL^−1^ from events μL^−1^ volume run for each of the five flow cytometry data types. For a total bacterial cell count, the SG HNA and LNA cell counts were then combined to form a total bacterial cell count in cells mL^−1^. For percent RSG count, the high RSG count was divided by the total bacterial cell count.

### 2.2 DNA Extraction and Amplicon Sequences

We used DNA to determine microbial community composition (via 16S/18S rRNA gene sequencing) closely following previous methods (Connors et al. 2024). In brief, 1 L of seawater was filtered through a sterile 0.2 μm Supor membrane disc filter (Pall Corporation, USA) and stored at −80°C until extraction. Filters were extracted using the KingFisher Flex Purification System and MagMax Microbiome Ultra Nucleic Acid Extraction kit (ThermoFisher Scientific, USA). For amplicon sequencing, extracted DNA was sent to Argonne National Laboratory for amplicon library preparation and sequencing using the Illumina MiSeq platform with the primers 515F and 806R for 16S rRNA sequencing (Walters et al. 2016) and 1380F and 1505R for 18S rRNA sequencing (Amaral‐Zettler et al. 2009) in a 2 × 151 bp library architecture.

Illumina reads were filtered, denoised and merged with DADA2 (Callahan et al. 2016) and then analysed with paprica v0.7.1 (Bowman and Ducklow 2015). Paprica utilises phylogenetic placement with Gappa (Czech et al. 2020), EPA‐ng (Barbera et al. 2019) and Infernal (Nawrocki and Eddy 2013), and RefSeq to place query reads on a reference tree constructed from the full‐length 16S rRNA genes from all completed genomes in GenBank (Haft et al. 2018) or 18S rRNA genes from all completed genomes in PR2 4.13.0 (Guillou et al. 2013). All unique reads were assigned to internal branches or terminal branches on the reference tree. Once assigned, unique reads that were assigned as metazoan mitochondria or chloroplasts were omitted, as well as any reads that only appeared once.

The paprica pipeline also includes use of the gRodon R package, a genomic estimator for maximal growth rate (cell doubling time, in hours) from codon usage patterns that has been adapted for use on 16S amplicon gene sequences (Weissman et al. 2021; Connors et al. 2024). We use gRodon to predict both individual amplicon sequence variant (ASV) predicted doubling time and mean doubling time for a given sample. These predictions are presented only in relative terms, without correction for temperature, as the estimate is a theoretical maximum growth rate based only on genetic signature and should only be interpreted in the relative sense for our data. To best reflect this, the gRodon mean doubling time is called a predicted relative mean minimal doubling time (PRMMDT) and the ASV predicted doubling time is a predictive relative minimal doubling time (PRMDT) moving forward in the text.

For our beta diversity analysis of the 16S rRNA and 18S rRNA gene amplicon sequences, unique reads were first cumulative sum scaled (CSS) to normalise for sampling depth across samples with the R package metagenomeSeq (Paulson et al. 2013). Then, non‐metric multidimensional scaling (NMDS) of Bray–Curtis distance and data dispersion of the CSS‐scaled relative abundance table and a calculation for the Shannon diversity index from the unscaled relative abundances were conducted with the vegan package (Oksanen et al. 2022). Post hoc analysis of variance in Bray–Curtis distances across months sampled was conducted with pairwiseAdonis (Martinez Arbizu 2020). Heatmaps of these data were created with the package pheatmap (Kolde 2019).

### 2.3 Induction Experiments

To determine the extent of lysogeny present during the season, we conducted once weekly viral induction experiments closely following previous methods (Paul and Weinbauer 2010; Brum et al. 2015). We subsampled 10 m water into six 50 mL sterile Falcon tubes and amended three with the inducing agent mitomycin‐C (final concentration 1 μg ml^−1^, Thermofisher, USA). Both the control and mitomycin‐C amended induction samples were incubated in the dark at in situ temperatures (2°C) for 24 h, and viral concentration was determined at the start and end of the incubation with flow cytometry as described above. Increases in viral concentration in the mitomycin‐C amended samples versus control samples (no treatment) were evaluated for statistical significance using ANOVA in R.

### 2.4 Dilution Experiments

**FIGURE 1:**
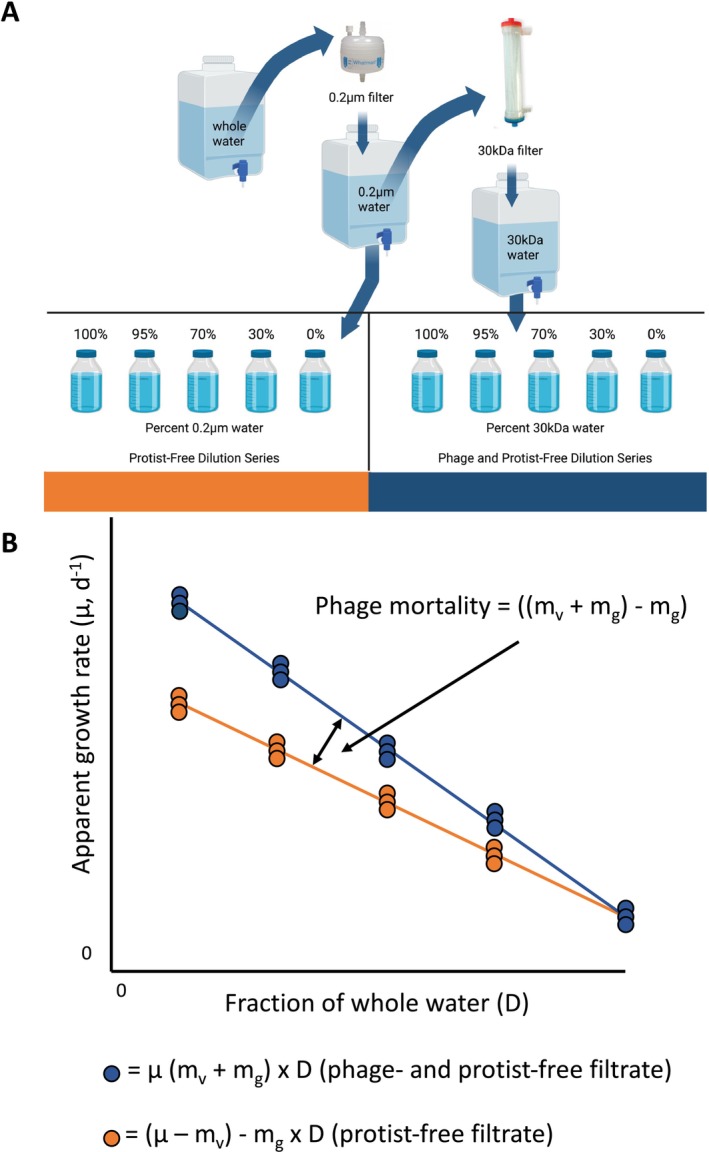
(A) Experimental set up and (B) hypothetical results of the dilution experiments. Seawater was filtered with 200 μm mesh (whole water), a 0.2 μm filter (protist‐free filtrate) and a 30 kDa filter (protist‐and‐phage free filtrate). Whole water was combined with either filtrate into two dilution series (either protist or combined, bottles represent triplicate samples). (B) Hypothetically, an increasing fraction of whole water over these experimental series should increase predator encounters and cause a decrease in bacterial growth rate over both dilution series, with the difference being the influence of phage predation.

We conducted 25 weekly dilution experiments through the two summer seasons to estimate and compare mortality rates of bacteria from either phages or protists following a modified version of previous methods (Kimmance and Brussaard 2010). We started with 30 L of seawater from 10 m and filtered the entire volume with a 200 μm filter into an acid‐washed and Type 1 water‐rinsed carboy to remove any large grazers from the whole seawater. We then passed half of the volume of whole seawater through a 0.2 μm filter (PALL, USA) to create protist‐free filtrate into an acid‐washed and rinsed carboy, and half of that filtered volume further through a 30 kDa filter (InnovaPrep, USA) to create protist‐and‐phage free filtrate in another acid‐washed and rinsed carboy. Dilutions were prepared by combining 0%, 30%, 70%, 95% and 100% of either type of filtrate with unfiltered seawater in triplicate 500 mL glass bottles (Figure 1A). Care was taken to maintain filtered and unfiltered seawater at < 4°C.

The protist‐free filtrate (protist dilution series) and virus‐free filtrate (combined dilution series) were incubated in the dark at in situ temperature (1.5°C) for 24 h. Samples for bacterial and viral abundance via flow cytometry as described above were taken at the start and end of the incubation from each bottle to determine bacterial growth rate over the experiment. Additionally, triplicate samples of whole seawater (500 mL each) were filtered for amplicon sequencing at the start of the experiment, and every bottle in the dilution series (500 mL) was filtered for amplicon sequencing at the end of the experiment to conduct an ASV‐level analysis of mortality during each experiment.

Apparent growth rates across these experiments are the changes in abundance during the incubation in each sample bottle using the following equation:

(1) $$\mu =\ln\left(P_{t}/P_{0}\right)/t$$

where *P*_*t*_ and *P*_0_ are the final and initial measured bacterial abundance, and *t* is the duration of the experiment (24 h).

Any apparent growth rates that were greater than two standard deviations from the other two replicates for each sample were discarded. Hypothetically, across either of the dilution series (0%–100% whole water), an increasing fraction of whole water should decrease the apparent growth rate of bacteria as the predators (depending on the dilution series, either just protists or protists and phages) are encountered at a higher rate. Therefore, a more negative change in growth rate over the dilution series indicates a higher predation pressure (Figure 1B). We also subtracted the change in growth rates in the combined dilution series (made with protist‐and‐phage free filtrate) from the only protist dilution series (made with just protist‐free filtrate) to determine the influence of phage mortality over an experiment. For our ASV‐level analysis of these dilution series, instead of using bacterial cell counts to calculate apparent growth rates, we used absolute abundance of each ASV (where the absolute abundance is the percent relative abundance of an ASV multiplied by total bacterial cell count) of every sample. Finally, to best compare each ASV's gRodon predicted relative minimal doubling time to the ASV‐level dilution experimental results, we calculated the change in apparent growth rate for both dilution series and calculated phage predation for every dilution experiment for the absolute abundance of each ASV (*n* = 25 for each ASV, as we did with bacterial cell counts). To best summarise these results, we calculated six averages for each ASV: averages of all negative growth rates for the three experimental conditions over all the dilution experiments, and averages of all positive growth rates for the three experimental conditions over all the dilution experiments. With these average values, we determined which taxa deviated from the mean ASV behaviour (either due to predation, via the negative slope data, or due to growth via the positive slope data) during our dilution experiments.

## 3 Results

### 3.1 Inter‐Seasonal and Intra‐Seasonal Changes in Temperature, Wind and Estimated Water Column Stratification

The PAL2223 and PAL2324 seasons had significantly different water temperature, air temperature, wind direction and estimated water column stratification, particularly in January–March. Water temperature was significantly higher in PAL2223 when compared to PAL2324, with temperatures reaching as high as 2.3°C on 12 February 2023 (ANOVA Month‐Year *p* = 2.2 × 10^−16^, Figure S1). Air temperature was also higher in January 2023 when compared to January 2024 (ANOVA Jan‐Year *p* = 1.3 × 10^−4^, Figure S2A), and wind direction was sustained in a predominantly easterly direction in only February and March 2023 during PAL2223 (ANOVA Month‐Year *p* = 3.1 × 10^−3^, Figure S2B).

To assess the impact of sustained wind direction in the later PAL2223 season, we estimated a proxy for water stratification by calculating the absolute value of the water temperature difference between 10 and 50 m (where a high number indicates strong water column stratification, Figure S3). While both seasons have the highest seasonal stratification in early January, estimated water column stratification is significantly lower in January 2023 (with a 0.8°C temperature difference) when compared to January 2024 (with a 1.2°C temperature difference, ANOVA Month‐Year *p* = 7.9 × 10^−5^, Figure S3A). The water column in February and March 2023 was also less stratified (with less than a 0.20°C difference between 10 and 50 m) than in February and March 2024 (with a more variable °C difference between 10 and 50 m, Figure S3A, ANOVA March‐Year *p* = 0.06).

### 3.2 Inter‐Seasonal and Intra‐Seasonal Changes in Cell Abundances

**FIGURE 2:**
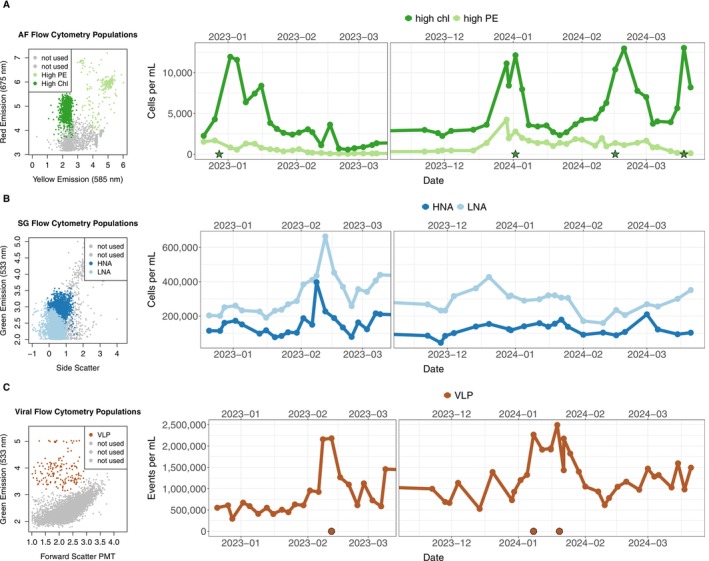
(A) Model flow cytometry output of AF sample with the high Chl and high PE populations highlighted, with the changes in cells per mL of the two populations over the two seasons to the right. (B) Model flow cytometry output of SG sample with the high nucleic acid (HNA) and low nucleic acid (LNA) populations highlighted, and the changes in cells per mL of the bacterial populations over the two seasons to the right. (C) Model flow cytometry output of viral sample with the viral like particles (VLP) population highlighted, and the changes in cells per mL of the VLP populations over the two seasons to the right. Green stars indicate experimental days with high chlorophyll values (> 10,000 cells mL^−1^) and maroon octagons indicate days with highest VLP values (> 2 million VLP mL^−1^).

Phytoplankton abundance, bacterial abundance, and VLP abundance all varied across each of the seasons and between the two seasons. High chlorophyll populations were highest in early January in both seasons, but only in PAL2324 were there additional high values in mid‐February and early March 2024 (ANOVA Month‐Year *p* = 6.6 × 10^−4^ for high Chl, Figure 2A). Bacterial abundances were also different over the months, with an anomalously high value in February 2023 (ANOVA Month‐Year *p* = 7.4 × 10^−3^ for HNA, Figure 2B). VLP abundance also varied each month but had their highest values at different times across the two seasons, in February 2023 and January 2024, respectfully (ANOVA Month‐Year *p* = 5.9 × 10^−14^ for VLP, Figure 2C), and values matched previous estimates (Brum et al. 2015). From our induction experiments, VLP counts from the mitomycin‐C amended samples were never significantly more abundant than the control samples, indicating a low level of lysogeny across the entire season (ANOVA *p* values for all experimental dates ranged from 0.08 to 0.92).

For PAL2223 the LSG‐stained samples showed that mixotrophic and heterotrophic cells were highest in early and mid‐January (ANOVA Month‐Year *p* = 1.0 × 10^−4^ for mixotrophic and 6.0 × 10^−4^ for heterotrophic cell abundance, Figure S4B). RSG stained samples indicated that bacterial activity was significantly different across the months of the two seasons, with the highest activity in February and March of 2024 (ANOVA Month‐Year *p* = 1.7 × 10^−3^ for log_10_(high RSG), Figure S5A). The high RSG cells in a sample were also positively log_10_‐correlated with gRodon PRMMDT (linear model adj *R* ^2^ = 0.31 and *p* = 9.9 × 10^−5^, Figure S5B).

### 3.3 Inter‐Seasonal and Intra‐Seasonal Changes in Community Composition

**FIGURE 3:**
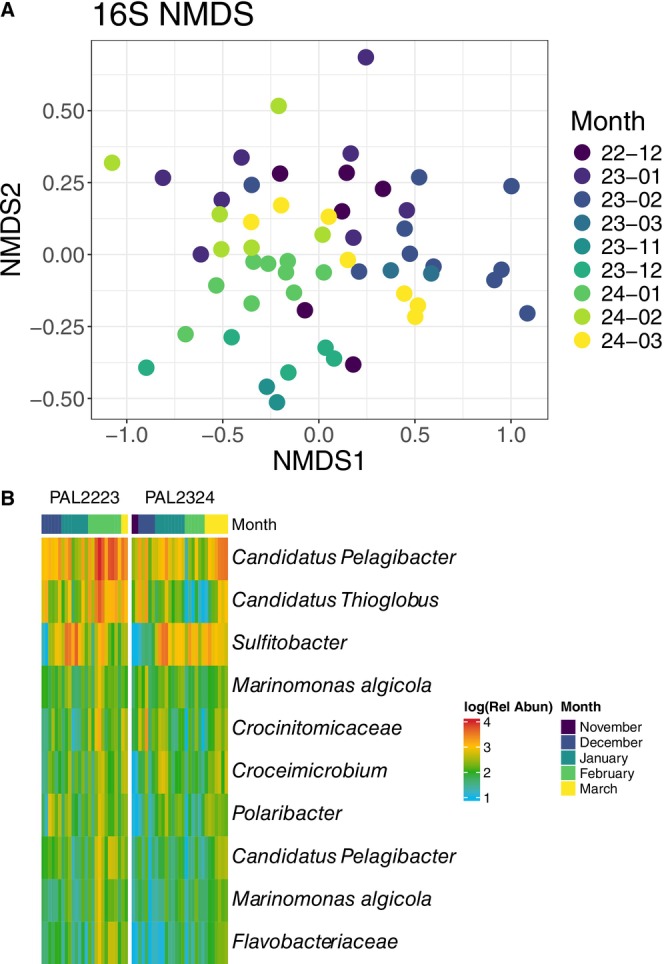
Relative abundance of 16S rRNA gene amplicon sequences across the season at 10 m. (A) is an NMDS plot (stress = 0.15) where the colour is month‐year. (B) Relative abundances of the 10 most highly abundant amplicon sequence variants (ASVs) over the season.

Community composition of bacteria and archaea from 16S rRNA gene sequencing and eukaryotes from 18S rRNA gene sequencing were also significantly different across months of the two seasons. In our NMDS analysis, Bray–Curtis distances of relative abundances were significantly different across the months of both seasons (ADONIS Month‐Year *p* value for 16S = 0.005, Figure 3A; 18S = 0.005, Figure S6A). The Bray–Curtis distances of relative abundances had significantly different dispersion across the seasons for both 16S and 18S data (Betadisper ANOVA Month‐Year *p* = 1.6 × 10^−5^ for 16S; *p* = 5.1 × 10^−4^ for 18S). Bacterial Shannon diversity had an average of 2.84 and did not significantly change across the months or years (ANOVA Month‐Year *p* = 0.298 for 16S). Eukaryotic Shannon diversity had an average of 3.12 and did not significantly change across the months (ANOVA Month‐Year *p* = 0.19 for 18S).

The most relatively abundant bacterial taxa included *Pelagibacter* and *Thioglobus*, which were more highly abundant in February and March of 2023, and *Sulfitobacter* which was the most highly abundant taxa in mid‐January of both years (Figure 3B). The absolute abundance of the two most abundant bacterial taxa, *Pelagibacter* and *Thioglobus* were highly correlated with our proxy for water stratification, with the highest abundances of these taxa present only in a poorly stratified water column (with < 0.6°C temperature difference between 10 and 50 m, Figure S2B).

The most relatively abundant eukaryotic taxon was the cryptophyte *Cryptomonadales*, which was especially dominant in January and February 2024 (Figure S6B). Unlike the dominant bacterial taxa *Pelagibacter*, *Cryptomonadales* had its highest relative abundance in the most highly stratified waters (> 0.9°C temperature difference between 10 and 50 m, Figure S2C). Other dominant taxa included the diatom *Thalassiosira* in early January of both seasons, dinoflagellates including *Gymnodiniaceae* and other unclassified dinoflagellates in January of both seasons but only in February and March of 2024 (Figure S6B).

### 3.4 Inter‐Seasonal and Intra‐Seasonal Predation Pressure From Dilution Experiments

**FIGURE 4:**
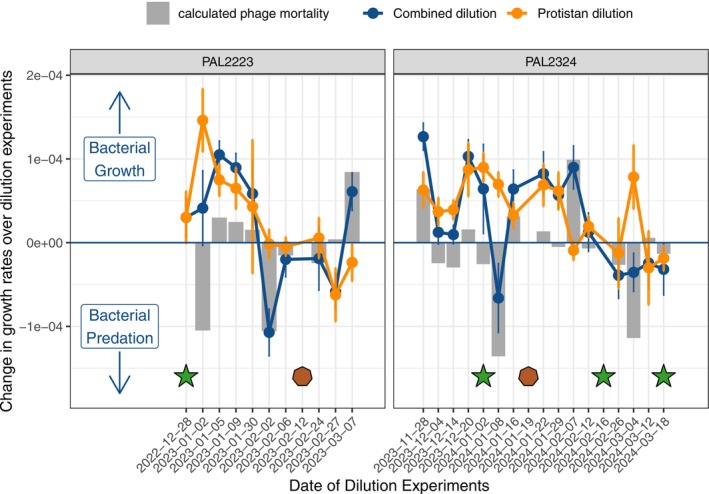
Dilution experiential results, indicating which experiments resulted in bacterial growth or bacterial predation. For each of the dilution experiments, a positive change in growth rate of either of the two dilution series (combined or protistan) indicates bacterial growth, while a negative change in growth rate over either of the dilution series (combined or protistan) indicates bacterial predation. The difference between change in growth rate over the two dilution series is the calculated phage mortality for each experiment (grey bars). Green stars indicate experimental days with high chlorophyll values (> 10,000 cells mL^−1^) and maroon octagons indicate days with highest VLP values (> 2 million VLP mL^−1^).

**FIGURE 5:**
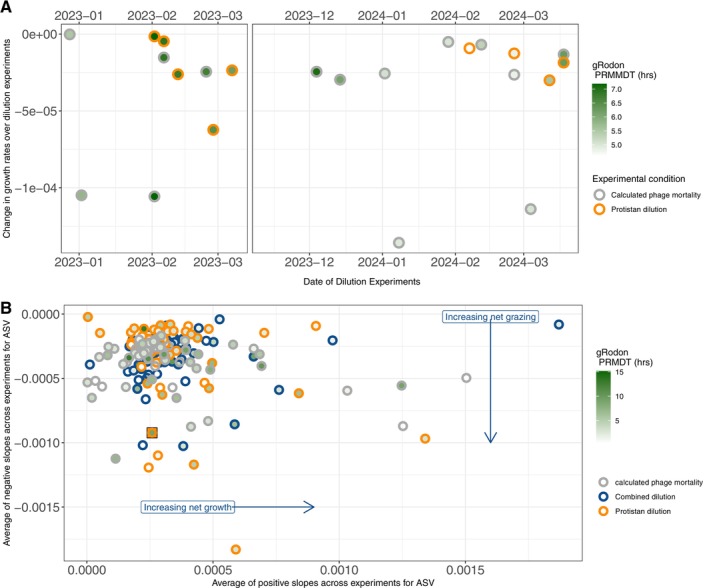
(A) Subset of dilution experimental results over time that resulted in bacterial predation (where the change in growth rate over the experiment was negative). Border colour indicates the experimental condition of the observation (protistan dilution series or calculated phage mortality). Colour of points indicates the gRodon predicted relative mean minimal doubling time (PRMMDT, in hours) of bacteria present in whole water before each experiment. (B) ASV‐level analysis of the dilution experiments. For each ASV, we show the average of all experimental results over time that resulted in bacterial predation (where the change in growth rate over the experiment was negative) versus the average of all experimental results over time that resulted in bacterial growth (where the change in growth rate over the experiment was positive). Border colour indicates the experimental condition of the observation (combined dilution series, protistan dilution series or calculated phage mortality) and colour of points indicates the gRodon predictive relative minimal doubling time (PRMDT) for each ASV, and the square point is the ASV assigned as *Pelagibacter*.

The PAL2223 and PAL2324 seasons followed similar patterns of protistan predation pressure as inferred from the dilution experiments. Our proxy for predation pressure, the change in the growth rate as a function of dilution (where a more negative slope indicates higher predation), decreased over the summer in both seasons (ANOVA Month‐Year *p* = 7.4 × 10^−3^, orange line in Figure 4). From this pattern in our experimental results, we can infer that bacterial growth outpaced predation from protists in the month of January but not in February or March for both seasons. The only date that was the exception to this pattern (with a highly positive change in the growth rate over the dilution experiment) was our dilution experiment from 4 March 2024, where bacterial growth outpaced protistan grazing immediately following the second phytoplankton bloom of the PAL2324 season when *Cryptomonadales* was a less dominant taxon and potential predator of bacteria (Figure 4; Figure S6). Interestingly, the dominance of protistan predation in February and March occurred even as the gRodon predicted relative mean minimal doubling time of the taxa was very divergent; samples in late summer 2023 had an average gRodon PRMMDT of 7 h while the samples in late summer 2024 had an average gRodon PRMMDT of 3 h (Figure 5A).

The pattern in our calculation for phage predation was also consistent across the two seasons, with the lowest calculated values for phage mortality (again, where a more negative number indicates predation pressure) directly following dates with high chlorophyll biomass (> 10,000 high chlorophyll cells mL^−1^, stars in Figure 4). The only exception to this is the negative phage mortality on 2 February 2023, which did not have a high chlorophyll biomass immediately preceding it. Finally, both the 2 February 2023 and 8 January 2024, phage predation events were directly followed by the highest value for VLPs (> 2 million VLP mL^−1^, maroon octagons in Figure 4).

### 3.5 ASV‐Level Analysis From Dilution Experiments

Although all bacteria demonstrated similar patterns in phage and protistan predation intra‐seasonally, our ASV‐level analysis indicates that specific genera have unique predation patterns that diverge from the overall pattern we found for all bacteria (where the most pronounced phage predation events followed phytoplankton blooms and protistan predation occurred in February and March for both seasons). Only a subset of the fast‐growing taxa demonstrated more negative average slopes across our dilution experiments (calculated as the average of all negative growth rates for a given experimental condition over all the dilution experiments for the ASV, Figure 5B). Interestingly, an ASV assigned as *Pelagibacter* was the only ASV that was slow‐growing (with a gRodon PRMDT of a 9‐h doubling time, square point in Figure 5B) that had more negative average slopes in the dilution experiments, indicating that it had high turnover despite being slow‐growing.

**FIGURE 6:**
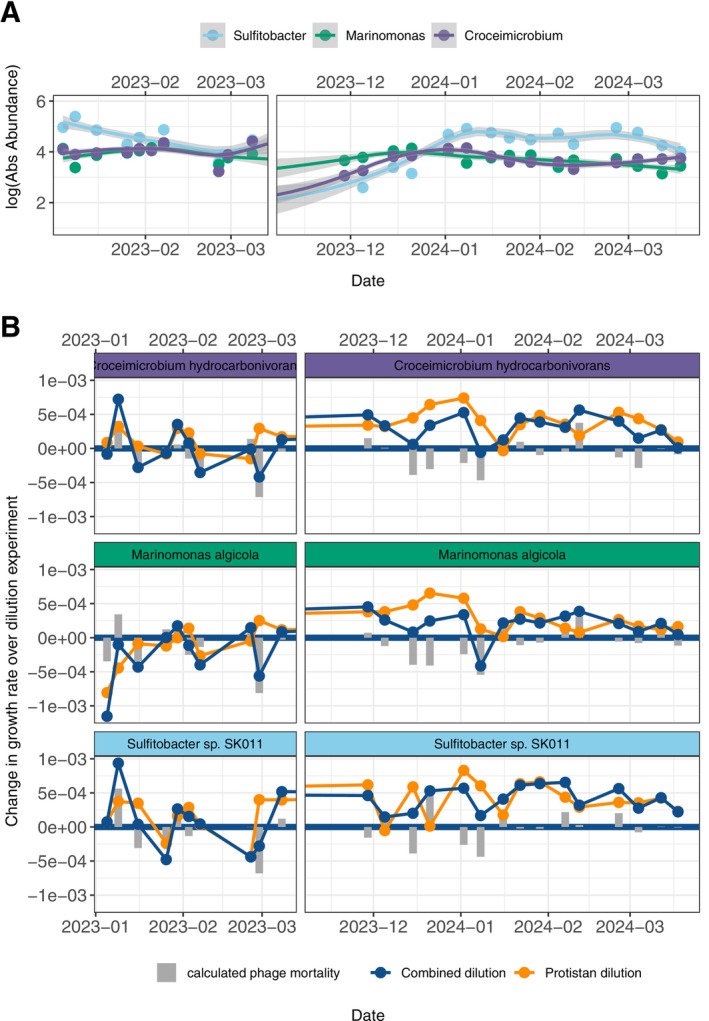
Log_10_(absolute abundance) of the three fastest growing most abundant taxa, *Sulfitobacter*, *Marinomonas* and *Croceimicrobium*. (B) ASV‐level dilution experimental results for each of the three fastest growing taxa. For each of the ASV‐level dilution experiments, a positive change in growth rate over either of the two dilution series (combined or protistan) indicates bacterial growth, while a negative change in growth rate over either of the dilution series (combined or protistan) indicates bacterial predation. The difference between change in growth rate over the two dilution series is the calculated phage mortality for each experiment (grey bars).

When we directly compared dilution experimental results across even ecologically similar taxa, there were clear differences in predation patterns across the two seasons for the different ASVs. This included ASV‐level dilution experiment results for the taxa with the fastest PRMDT from gRodon of the Top 10 most abundant ASVs (Figure 6, all three with gRodon PRMDT < 4 h). For instance, *Marinomonas algicola* had many more phage predation events during the dilution experiments of PAL2223, while the ASV *Sulfitobacter* demonstrated more protist predation events in January and February of PAL2324 when comparing their ASV‐level dilution experiment results directly over the two seasons (Figure 6B).

## 4 Discussion

We performed 25 dilution experiments over two highly variable summers at Station E, near Palmer Station, Antarctica. Our results show that KtW cannot be broadly applied to fast‐growing bacterial taxa, but that protists and phage do selectively target some fast‐growing or abundant bacteria which we term ‘kill select winners’ (KsW). First, our results clearly demonstrated the expected seasonal succession of rapid phytoplankton growth (with an assumed increase in dissolved organic carbon), followed by increased growth of heterotrophic bacteria, and finally predation of the rapidly growing bacteria, first primarily by phages and then by protists. The two seasons experienced similar intra‐seasonal predation patterns despite significant differences in hydrographic conditions and dominant taxa in late summer. Critically, a subset of the fastest growing bacteria (with the lowest predicted doubling time, in hours) and the slow‐growing taxon *Pelagibacter* experienced the highest level of activity in our dilution experiment results, demonstrating KtW dynamics for both protist and phage predators in this marine environment.

The summer season dynamics of coastal Station E, Antarctica began with a mixed phytoplankton bloom of the diatom *Thalassiosira* and dinoflagellates like *Gymnodiniaceae* by the first week of January in both seasons. These eukaryotic genera are very typical of the region, and the timing and magnitude (~12,000 chlorophyll containing cells mL^−1^, Figure 1) of these phytoplankton blooms are also typical for waters near Palmer Station, especially in recent years (Bowman et al. 2021; Ducklow et al. 2022; Nardelli et al. 2023; Turner et al. 2024). Interestingly, we also saw our highest levels of mixotrophic cells (those containing chlorophyll and positively stained by Lysotracker Green, Figure S4) during this early season bloom in PAL2223. Mixotrophy in polar seas has been hypothesized to be most important during early spring and fall when light is less available, a time period we did not take measurements for in our study (Stoecker and Lavrentyev 2018). Further study, especially on longer time scales including the very early spring and late fall, is necessary to better understand the seasonal succession of mixotrophs and the importance of mixotrophic protists for bacterial top‐down control in polar waters and marine environments more broadly.

In both seasons, the early phytoplankton bloom was immediately followed by a phage predation event in our next dilution experiment (with highly negative values calculated for phage mortality in the dilution experiment, Figure 4). In both seasons, the highly abundant and quickly growing (with a gRodon PRMDT estimated at 1.6 h) taxa *Marinomonas algicola* was especially vulnerable to phage predation in the ASV‐level analysis of these dilution experiments (with highly negative values calculated for phage mortality following phytoplankton blooms in the ASV‐level analysis, grey bars in Figure 6B). The rapid growth of *Marinomonas* directly following phytoplankton blooms is not surprising, as it is a known phytoplankton‐associated taxa and carbon degrader (Zhang et al. 2023). The phage mortality event is also supported by recent viral metagenomic work in the region, where a few dominant bacteriophages accounted for a substantial portion of increased viral activity in the early summer (Piedade et al. 2024).

Other taxa that were less abundant overall than *Marinomonas* but shared the same susceptibility to phage attack in the dilution experiments were *Oceanospirillales* (gRodon PRMDT estimated at 3.2 h), *Planktomarina temperate* (gRodon PRMDT 4.0 h) and *Kordia* (gRodon PRMDT 1.9 h). These taxa are all ecologically similar to *Marinomonas* as well, as all are known to associate with phytoplankton and rapidly grow in environments with high dissolved organic carbon (Beyer et al. 2016; Liu et al. 2023). The ecological role of *Kordia* is especially interesting as it is algicidal to certain phytoplankton, including bloom‐forming diatoms (Bigalke et al. 2019). Overall, our study indicates that phytoplankton‐associated bacterial taxa are highly susceptible to phage attack in early January, with important implications for the fate of bacterial carbon in the microbial loop. This viral shunt we observed, where bacterial biomass was most likely returned to dissolved organic carbon via phage attack (Wilhem and Suttle 1999), prevents a strong microbial loop from forming and carbon moving to higher trophic levels via protistan predation, which was not dominant until later in the summer.

In both PAL2223 and PAL2324, the phytoplankton blooms and phage mortality events of the early summer were succeeded by the rapid proliferation of the heterotrophic bacteria *Sulfitobacter* by mid‐January. *Sulfitobacter* has been previously identified as a successor to phytoplankton blooms in the region and was the most important taxa for predicting high bacterial production (the uptake of dissolved organic carbon by bacteria) in previous works near Palmer Station (Bowman et al. 2017; Connors et al. 2024). In our ASV‐level analysis of the dilution experiments from both seasons, *Sulfitobacter* also demonstrated a high susceptibility to protistan predation during December and January (Figure 6B). We hypothesise that the protistan grazing of *Sulfitobacter* may have been carried out by the dominant eukaryotic taxa *Cryptomonadales*, which demonstrated high relative abundance in the most highly stratified waters of January in both seasons.

While the two seasons varied hydrographically in February and March, we observed similar predation patterns in our dilution experiments. PAL2223 was dominated by sustained easterly winds, low water column stratification (Figure S3) and the dominance of the bacterial genera *Pelagibacter* and *Thioglobus*. The increase in *Thioglobus* at 10 m especially is an indication that the wind event increased vertical mixing in the water column, as it is usually found in deeper water (Shah et al. 2019; Dutta et al. 2023). In contrast, PAL2324 had more variable winds and water column stratification (Figure S3) which supported two additional phytoplankton blooms in February and March of 2024 (Figure 2). Despite their differences, both seasons demonstrated protistan predation pressure (negative change in growth rate over the dilution experiments, Figure 5A) in February and March. The large (> 50 μm) dinoflagellate *Gymnodiniaceae* and other unclassified dinoflagellates were present in high relative abundance (Figure S6) and, as known mixotrophs, may have been responsible for the observed predation (Doblin et al. 2004; Jeong et al. 2018). The later season dominance of larger cells was only seen in our 18S rRNA gene amplicon sequencing, as our flow cytometers could only count cells ranging from 0.02 to 40 μm. The lack of cell abundance information for larger cells is a shortcoming of our study, as it is difficult to estimate the contribution of larger cells such as *Gymnodiniaceae* to bacterial predation without cell density information. Additionally, while it would be ideal to use our 18S taxon assignments to assign feeding modalities, potential for parasitism, or even bottom up competitive advantages to the protists, as has been done in other environments (Coats 1999; Jones 2001; Ramond et al. 2019), a high degree of uncertainty for polar protists trophic modes persists. A better understanding of trophic modes is necessary, as protistan predators can show preferences for certain prey but also take up the prey they are the most likely to encounter, which may have contributed to the observed predation pattern (Weisse et al. 2016). Future work must include metagenomic and culture‐based work to determine the true diversity and function including the prey preference of polar protists, especially the largely unclassified dinoflagellates.

While our intra‐seasonal analysis primarily demonstrates the complexity of environmental microbial communities, we did observe direct evidence of KtW in a natural assemblage of microbes, though it did not apply to all abundant or fast‐growing taxa. Of the bacterial ASVs that experienced the highest level of predation (the Top 30 ASV‐level predation events), a subset of the faster growing microbes experienced higher levels of predation by both protists and phages in our dilution experiments (Figure 5B). Interestingly, those bacteria that experienced the highest levels of predation and demonstrated KtW were only a subset of the fast‐growing taxa that were present across the season, as many of the bacteria with < 5 h predicted growth rates experienced only moderate activity (0–0.01 ASV activity, Figure 5B). Our findings indicate that both a fast growth rate and a population threshold may need to be reached for excessive phage lysis, as previous modelling work has indicated (Maslov and Sneppen 2017). Overall, our findings highlight the importance of time series predation data to interpret results from a mixed environmental community, as bacterial growth and predation rates can change very rapidly.

Our study demonstrates clear seasonal succession and KtW dynamics of the microbes in a coastal marine station near Palmer Station, Antarctica. Over both summers, an early summer phytoplankton bloom resulted in a rapid phage attack of fast‐growing phytoplankton‐associated bacteria including *Marinomonas*. This was followed by a bloom of the heterotrophic bacteria *Sulfitobacter* and the eukaryotic mixotroph *Cryptomonadales*, which may have contributed to the protistan predation of *Sulfitobacter* that we observed in January. In February and March, large dinoflagellates dominated bacterial predation, even when the dominant bacterial species was the slow‐growing *Pelagibacter* in PAL2223. Our evaluation of bacterial abundance and community structure over the austral summer provides unprecedented knowledge of top‐down controls for Antarctic marine bacteria, and we successfully observed selective KtW dynamics in a natural assemblage of microbes.

## Author Contributions

**Jeff S. Bowman:** conceptualization, funding acquisition, methodology, investigation, project administration, supervision, data curation, software, formal analysis, writing – review and editing. **Abigail Coker:** writing – review and editing, investigation, data curation. **Elizabeth Connors:** writing – original draft, visualization, formal analysis, software, data curation, validation, project administration, investigation. **Grace S. Wang:** writing – review and editing, investigation, data curation.

## Funding

This study was supported by an NSF CAREER award to J.S.B. (NSF OPP‐1846837), and the Palmer Station Long Term Ecological Research (PAL‐LTER) is supported by the National Science Foundation Office of Polar Programs (NSF OPP‐2026045).

## Conflicts of Interest

The authors declare no conflicts of interest.

## Supporting information


**Figure S1:** Interpolated changes to water temperature over the two seasons (A) PAL2223 and (B) PAL2324. Black points are sampling points over the months and depths sampled.

**Figure S2:** (A) Average wind speed (in km h^−1^) with wind direction and (B) minimum air temperature (in °C) measured every day over the two seasons at Palmer Station, Antarctica.

**Figure S3:** Water column stratification (A) °C difference between 10 and 50 m over time, where the highest stratification occurs in January. (B) Absolute abundance (cells per mL) of *Pelagibacter* and *Thioglobus* over °C difference between 10 and 50 m. (C) Relative abundance (cells per mL) of *Cryptophyta* over °C difference between 10 and 50 m. Colour is total Chl cells per mL from flow cytometry.

**Figure S4:** (A) Model flow cytometry output of AF sample, with the high chlorophyll and high PE populations highlighted, with the changes in cells per mL of these two populations over the season to the right. (B) Model flow cytometry output of LSG sample with the heterotrophic and mixotrophic populations highlighted, and the changes in cells per mL of these populations over the season to the right.

**Figure S5:** Model flow cytometry output of RSG sample, with the high RSG populations highlighted, with high RSG populations over the seasons to the right. (B) log_10_(high RSG) is linearly negatively correlated to average gRodon PRMMDT (adj *R* ^2^ = 0.3).

**Figure S6:** Relative abundance of 18S rRNA gene amplicon sequences across the season at 10 m. (A) NMDS plot (stress = 0.15) where the shape of point is depth (10, 30 and 50 m) and colour is month. (B) Relative abundances of the 10 most highly abundant 18S gene amplicon sequence variants (ASVs) over the season.

**Table S1:** Results from every dilution experiment, including the change in growth rate (mortality) from the combined dilution series and the error, the mortality from the protist dilution series and the error and the calculated influence from phages (phage mortality) and the error.

**Table S2:** ASV dilution experiment results, including the change in growth rate (mortality) from the combined dilution series, the mortality from the protist dilution series and the calculated influence from phages (phage mortality).

## Data Availability

Code and data repository for this manuscript is located on the first author's GitHub. Raw flow cytometry files are located at the US Antarctic Program Data Center (https://www.usap‐dc.org/view/dataset/601930). All sequences are available at NCBI SRA BioProject PRJNA901488.
