# Supplementary figures and images for: An Explicit Test of Kill the Winner: Protistan Grazing and Phage Lysis Differentially Impact Fast‐Growing Bacterial Taxa in the Coastal Antarctic
Source: Environ Microbiol. 2026 Feb 9;28(2):e70254. doi: 10.1111/1462-2920.70254 (PMC12886177; doi:10.1111/1462-2920.70254)

**A** Station E Temperatures over PAL2223

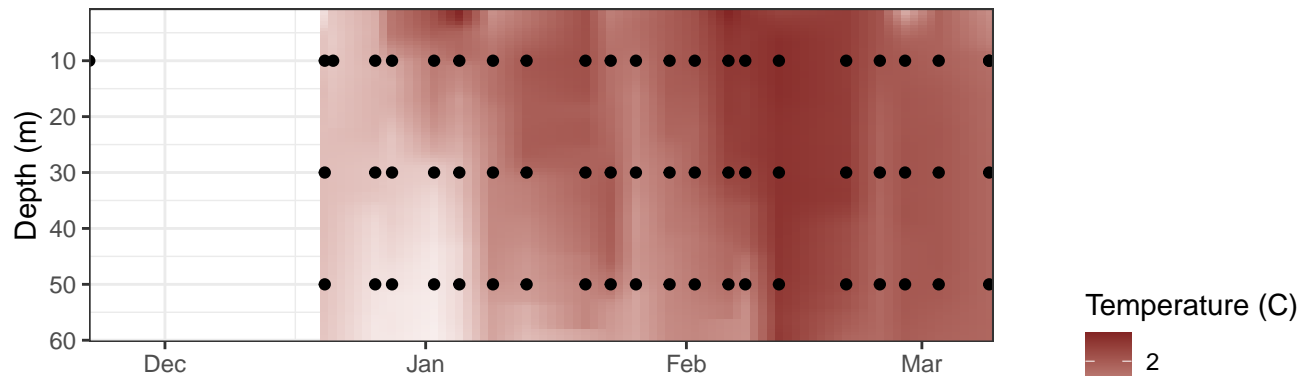

**B** Station E Temperatures over PAL2324

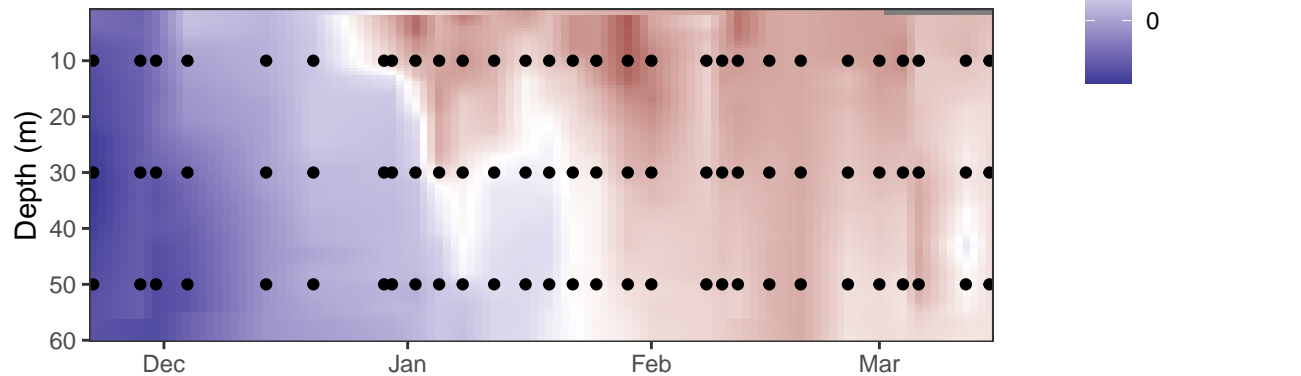

Supplement: Supplementary file 1 — Figure S1: Interpolated changes to water temperature over the two seasons (A) PAL2223 and (B) PAL2324. Black points are sampling points over the months and depths sampled. [file EMI-28-e70254-s005.pdf]

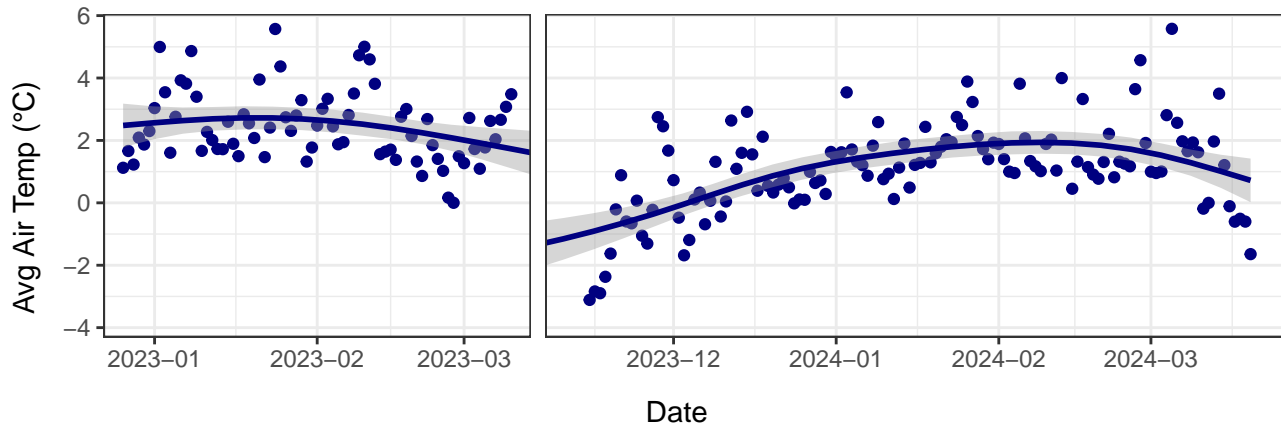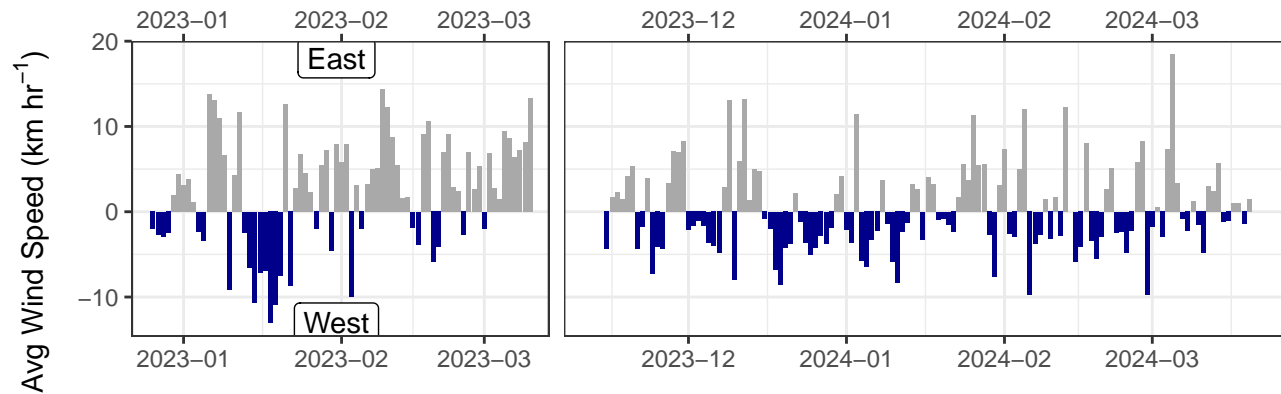

Supplement: Supplementary file 2 — Figure S2: (A) Average wind speed (in km h−1) with wind direction and (B) minimum air temperature (in °C) measured every day over the two seasons at Palmer Station, Antarctica. [file EMI-28-e70254-s004.pdf]

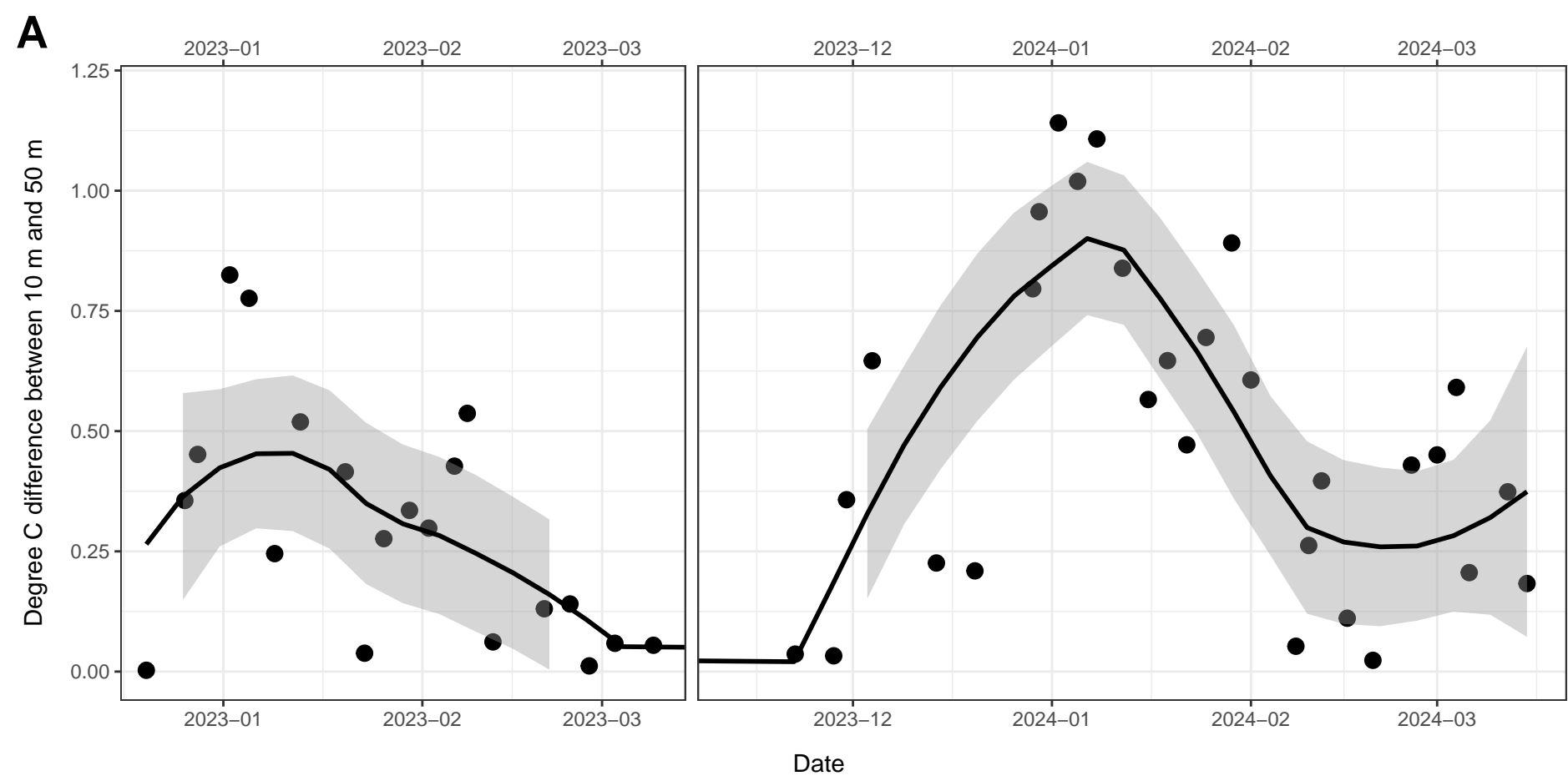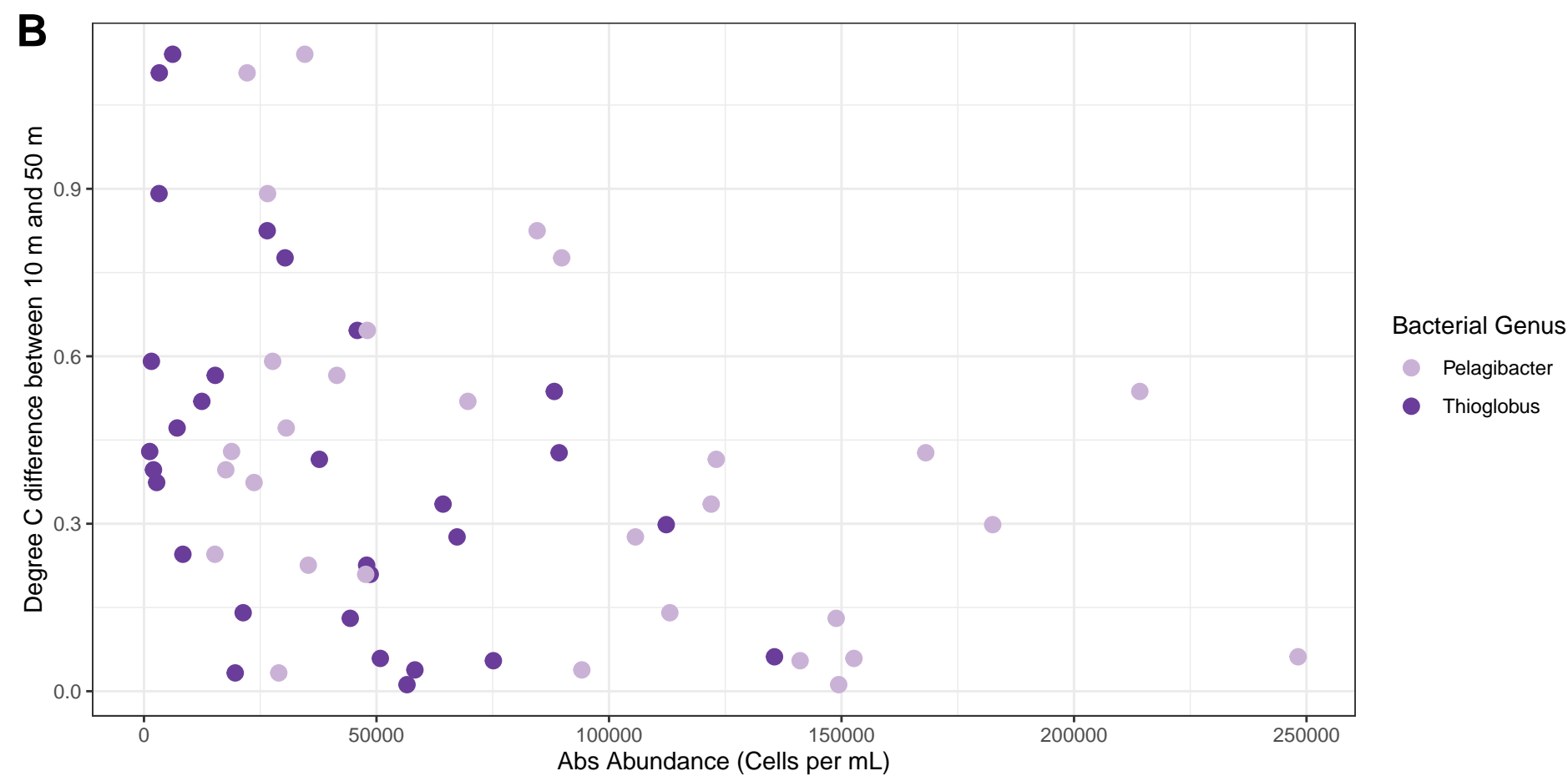

Supplement: Supplementary file 3 — Figure S3: Water column stratification (A) °C difference between 10 and 50 m over time, where the highest stratification occurs in January. (B) Absolute abundance (cells per mL) of Pelagibacter and Thioglobus over °C difference between 10 and 50 m. (C) Relative abundance (cells per mL) of Cryptophyta over °C difference between 10 and 50 m. Colour is total Chl cells per mL from flow cytometry. [file EMI-28-e70254-s007.pdf]

**A****AF Flow Cytometry Populations**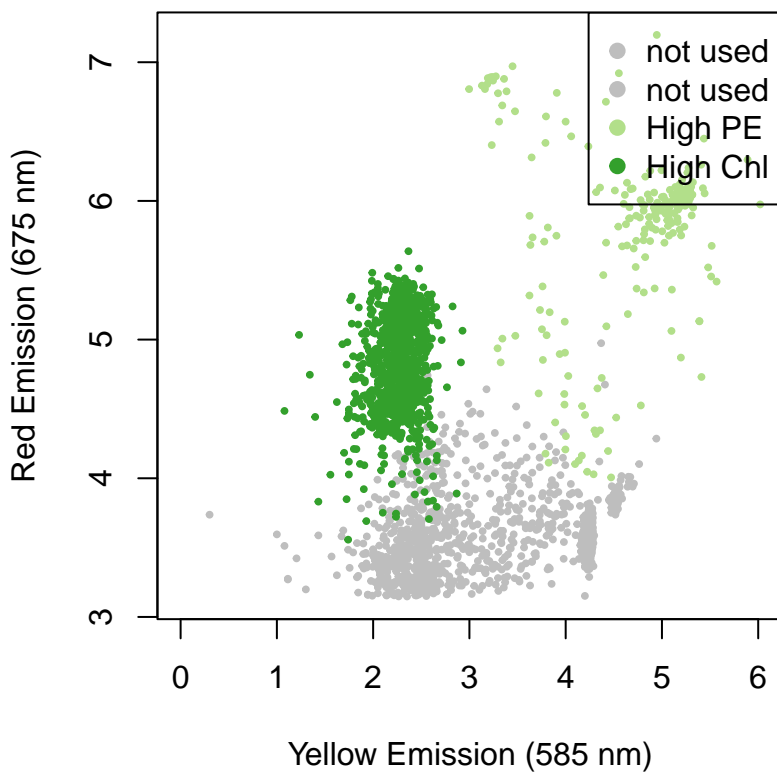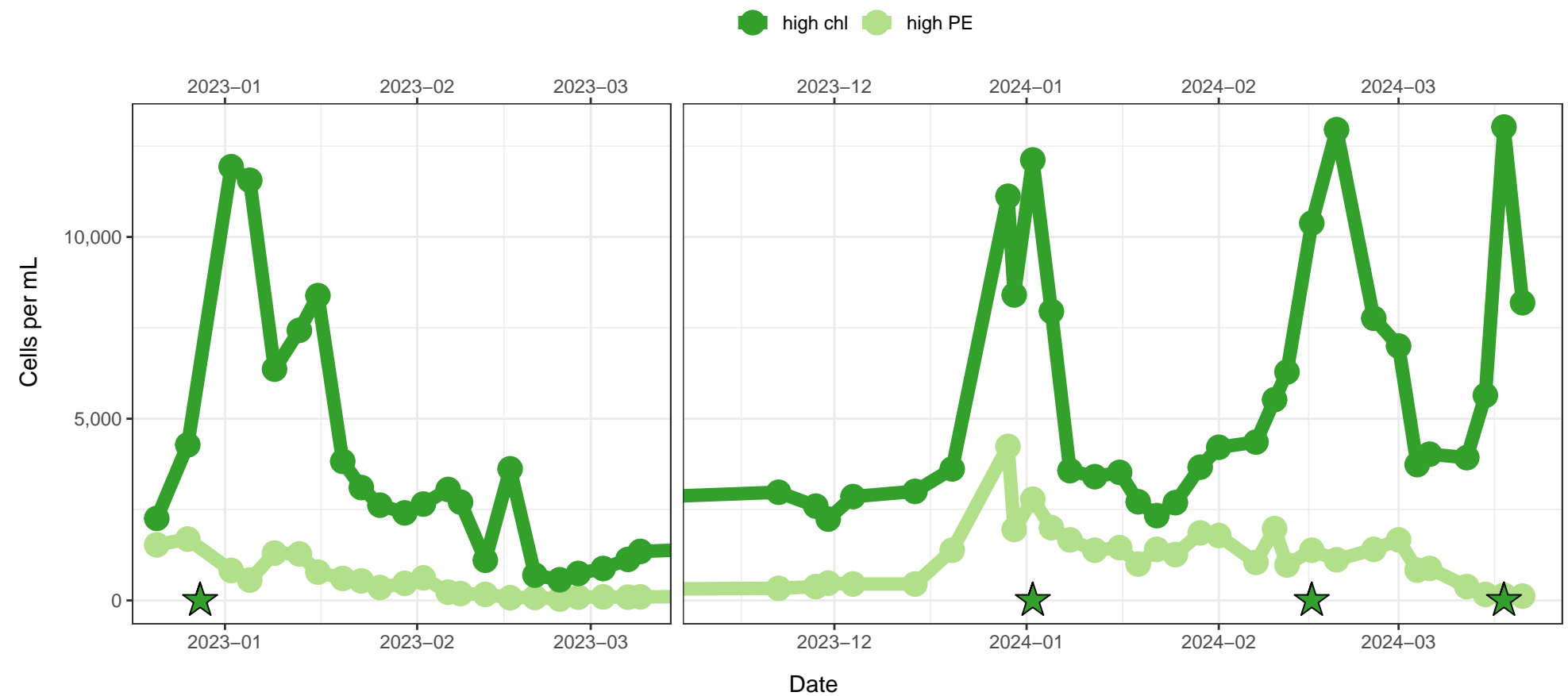**B****Lysotracker Flow Cytometry Populations**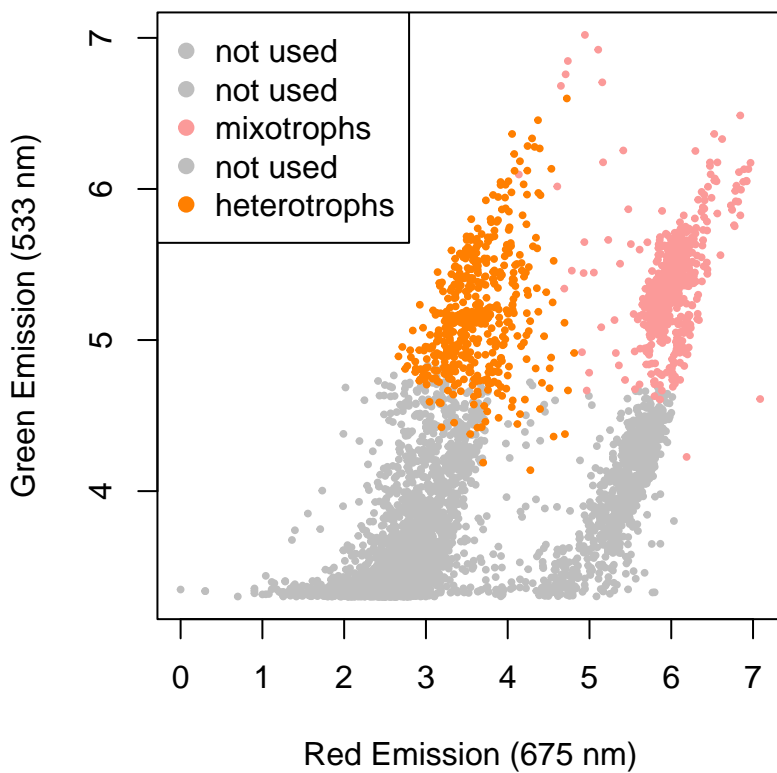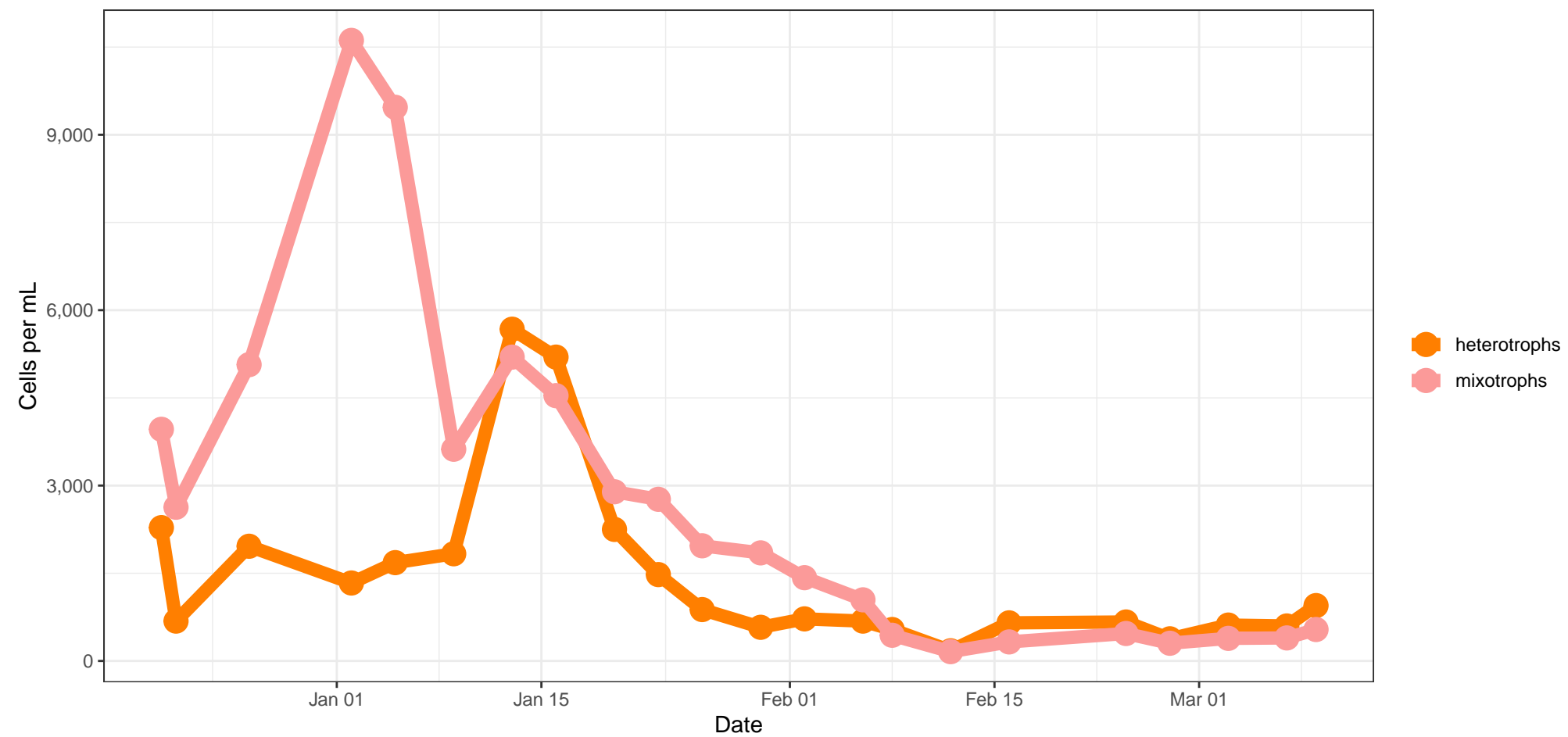

Supplement: Supplementary file 4 — Figure S4: (A) Model flow cytometry output of AF sample, with the high chlorophyll and high PE populations highlighted, with the changes in cells per mL of these two populations over the season to the right. (B) Model flow cytometry output of LSG sample with the heterotrophic and mixotrophic populations highlighted, and the changes in cells per mL of these populations over the season to the right. [file EMI-28-e70254-s001.pdf]

**A****RSG Flow Cytometry Populations**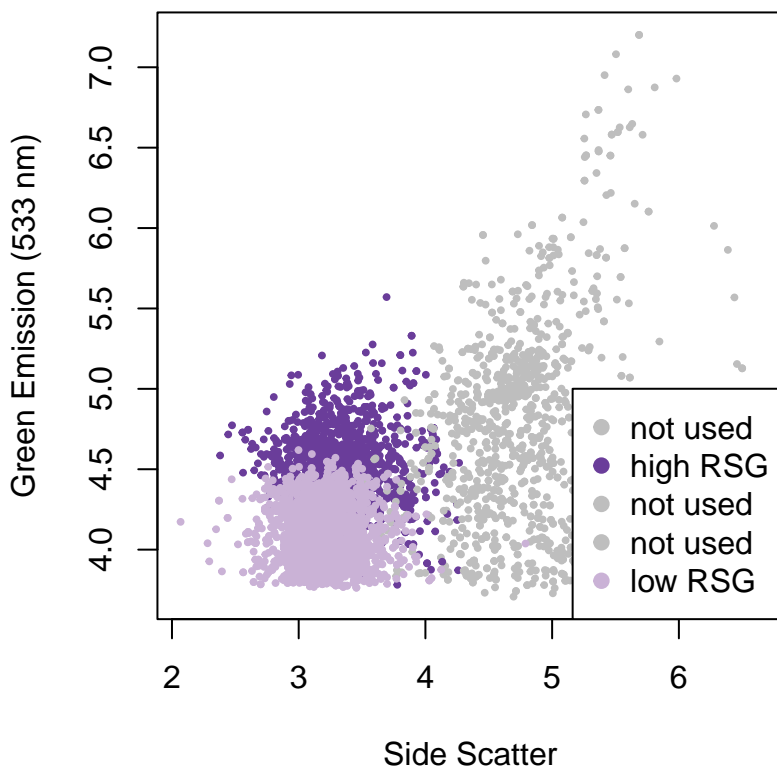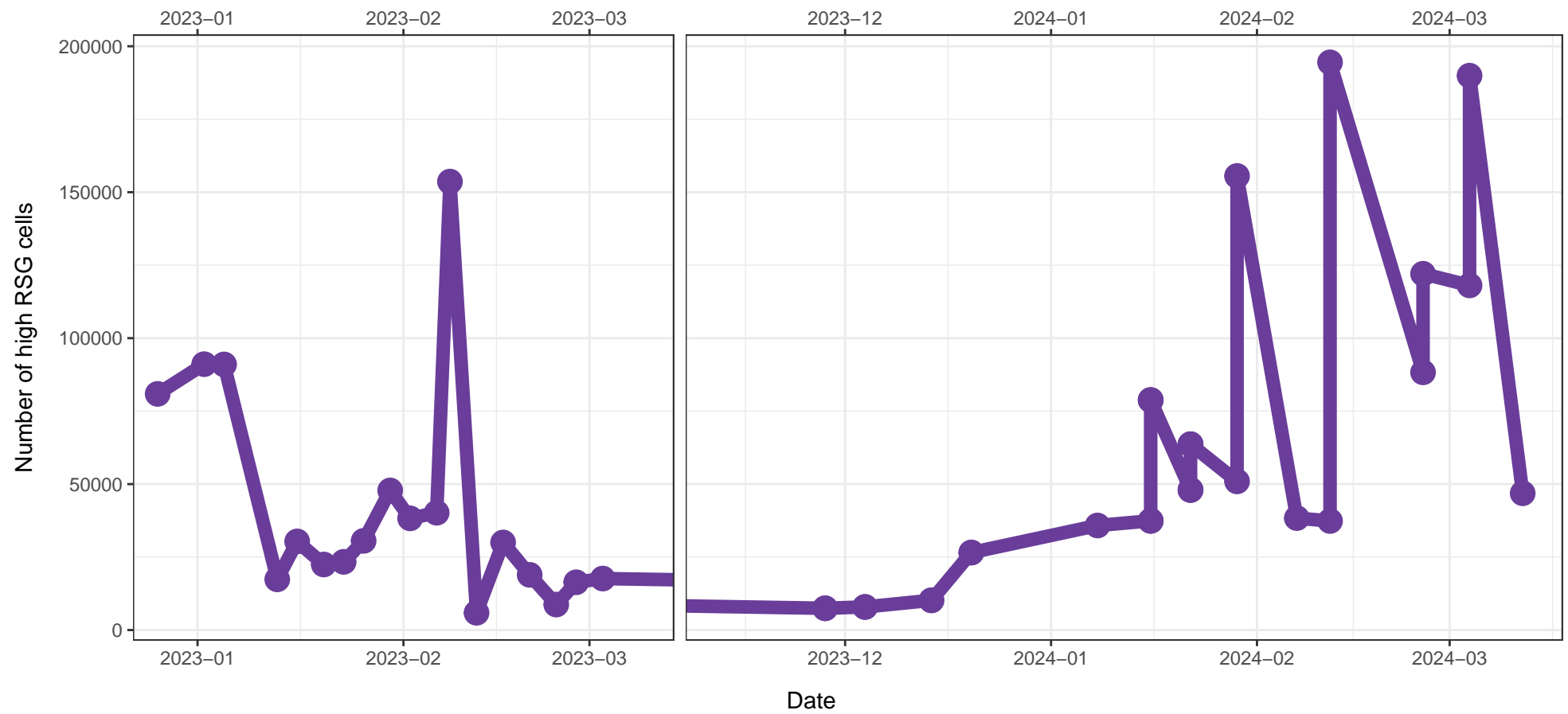**B**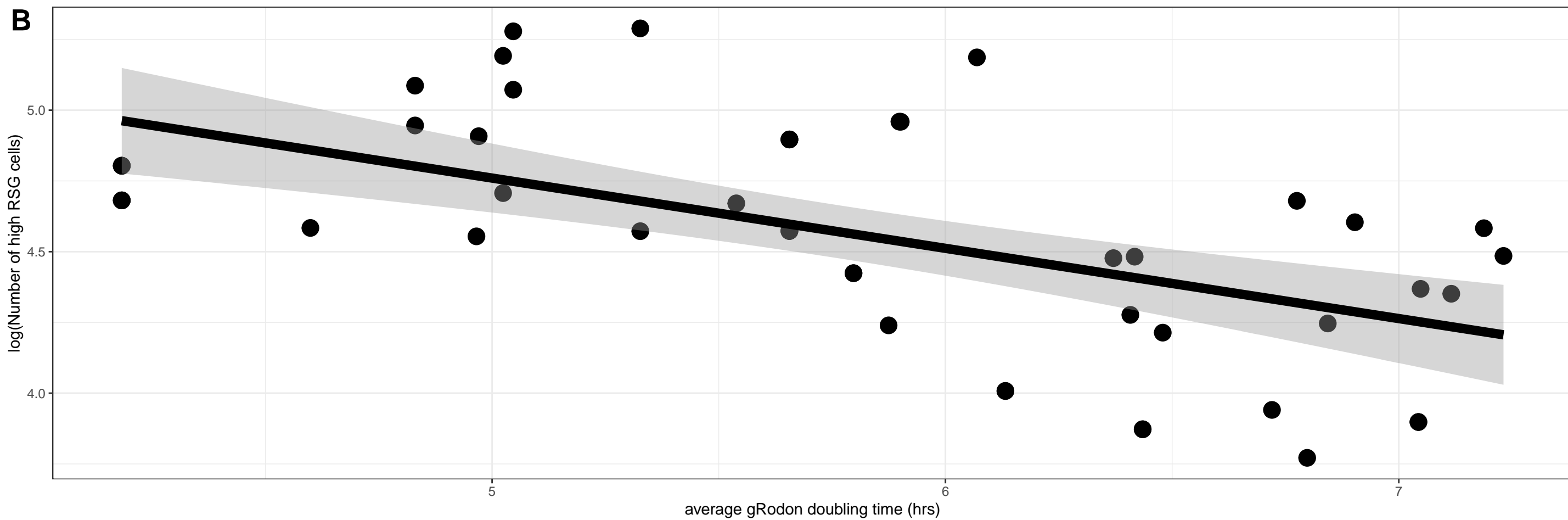

Supplement: Supplementary file 5 — Figure S5: Model flow cytometry output of RSG sample, with the high RSG populations highlighted, with high RSG populations over the seasons to the right. (B) log10(high RSG) is linearly negatively correlated to average gRodon PRMMDT (adj R 2 = 0.3). [file EMI-28-e70254-s006.pdf]

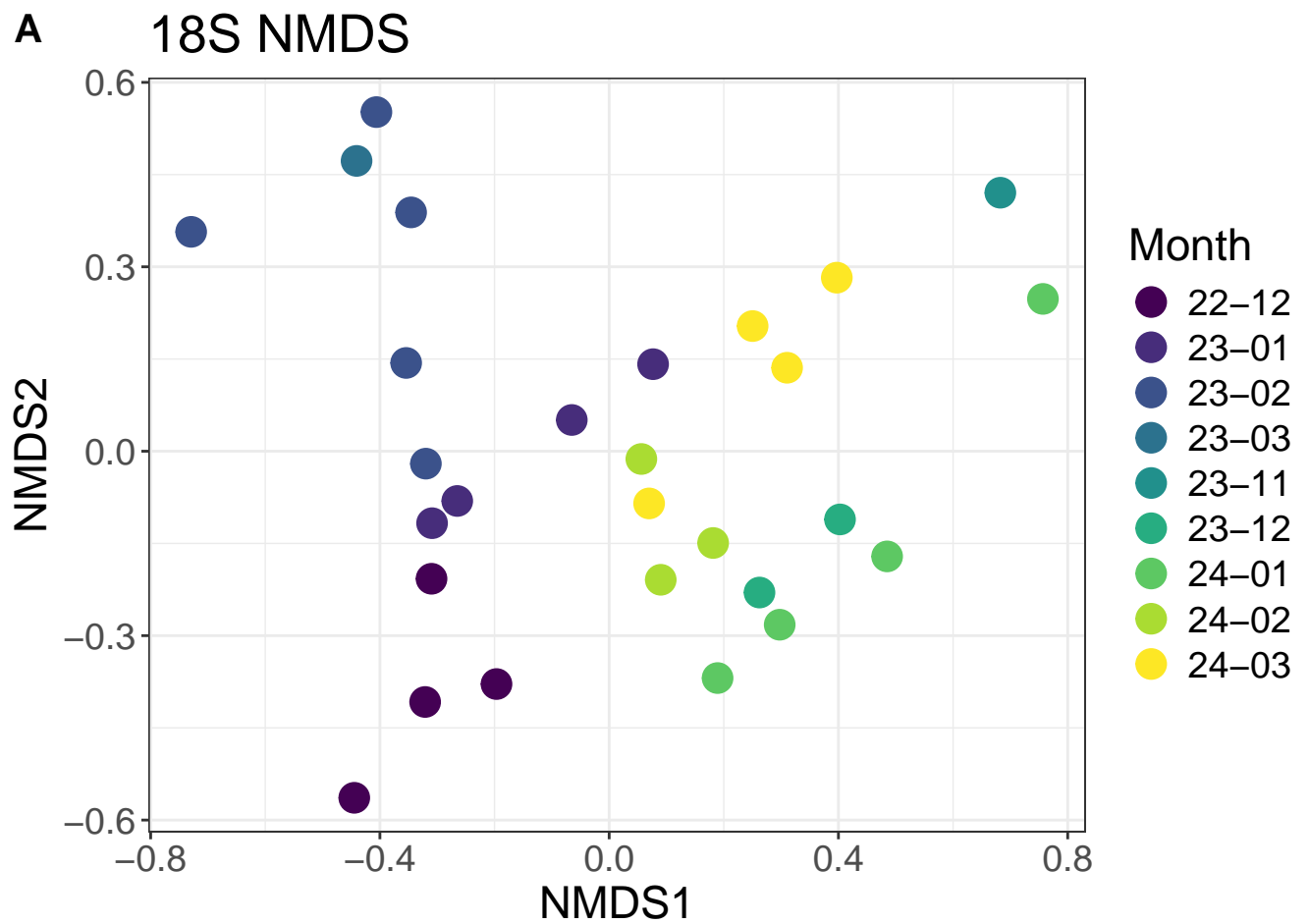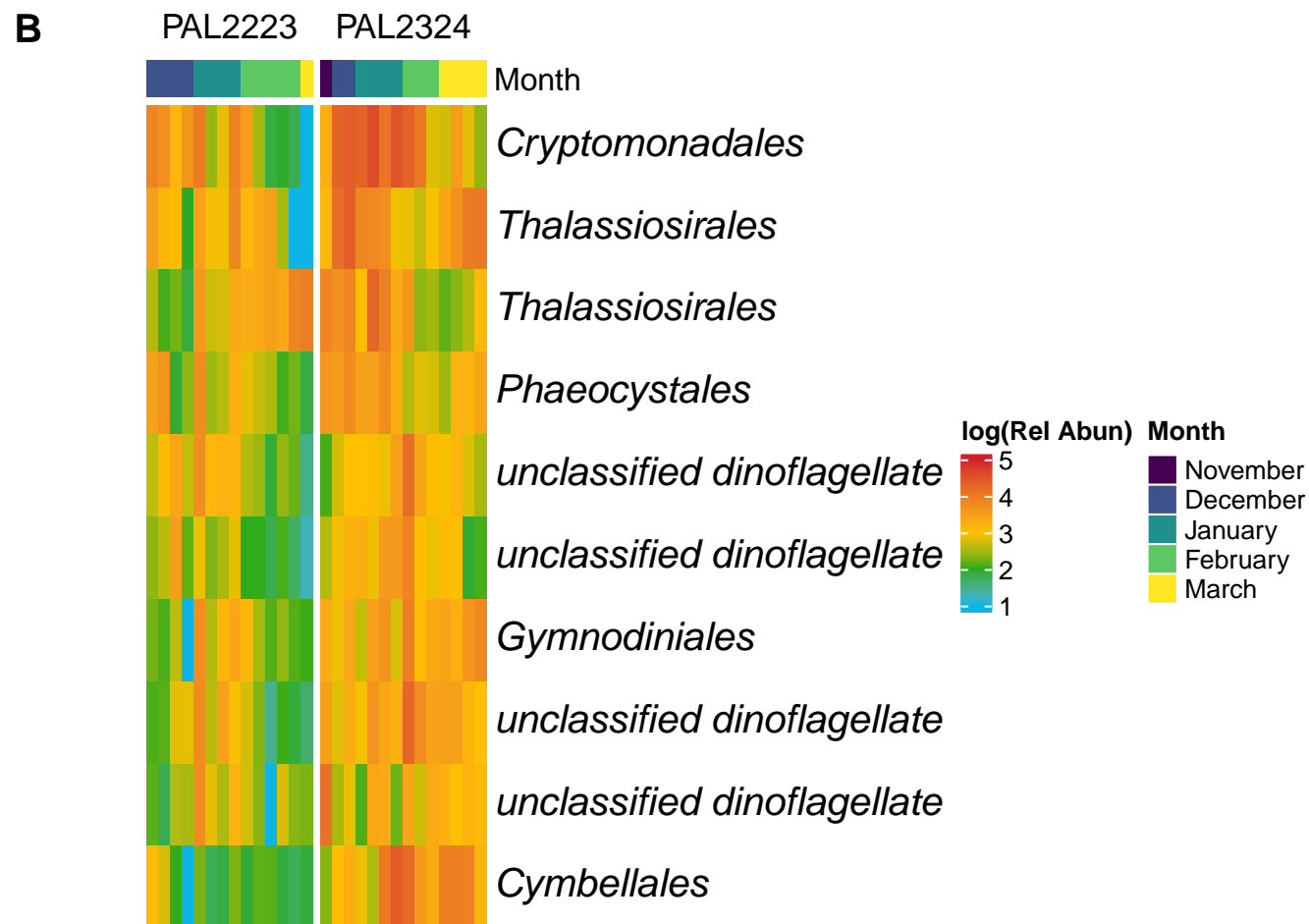

Supplement: Supplementary file 6 — Figure S6: Relative abundance of 18S rRNA gene amplicon sequences across the season at 10 m. (A) NMDS plot (stress = 0.15) where the shape of point is depth (10, 30 and 50 m) and colour is month. (B) Relative abundances of the 10 most highly abundant 18S gene amplicon sequence variants (ASVs) over the season. [file EMI-28-e70254-s008.pdf]
